# Voxel-based cortical thickness measurements in MRI

**DOI:** 10.1016/j.neuroimage.2008.01.027

**Published:** 2008-05-01

**Authors:** Chloe Hutton, Enrico De Vita, John Ashburner, Ralf Deichmann, Robert Turner

**Affiliations:** a Wellcome Trust Centre for Neuroimaging, Institute of Neurology, UCL, London, UK; b Department of Medical Physics and Bioengineering, UCL Hospitals NHS Foundation Trust, London, UK; c University Hospital, ZNN, Brain Imaging Center, Frankfurt, Germany; d Max Planck Institute for Human Cognitive and Brain Sciences, Leipzig, Germany

## Abstract

The thickness of the cerebral cortex can provide valuable information about normal and abnormal neuroanatomy. High resolution MRI together with powerful image processing techniques has made it possible to perform these measurements automatically over the whole brain. Here we present a method for automatically generating voxel-based cortical thickness (VBCT) maps. This technique results in maps where each voxel in the grey matter is assigned a thickness value. Sub-voxel measurements of thickness are possible using sub-sampling and interpolation of the image information. The method is applied to repeated MRI scans of a single subject from two MRI scanners to demonstrate its robustness and reproducibility. A simulated data set is used to show that small focal differences in thickness between two groups of subjects can be detected. We propose that the analysis of VBCT maps can provide results that are complementary to other anatomical analyses such as voxel-based morphometry.

## Introduction

Measuring the thickness of the human cerebral cortex is of great interest in studies of both normal and abnormal neuroanatomy. The average thickness over the whole brain is around 2.5 to 3 mm and within individual brains varies from about 2 mm at its thinnest in the calcarine cortex up to 4 mm and over in the thicker regions of the precentral gyrus, superior frontal lobes and superior temporal lobes (Zilles, 1990). These variations relate to differences in cell types (Geyer et al., 1999) and may also be associated with functionally distinct areas (Brodmann , 1908; Von Economo and Koskinas, 1925). Studies have also shown that the cortex is thicker at the gyral ridges and thinner at the fundi of the sulci. Although cortical thickness varies between individuals, abnormally thick or thin cortex may be associated with changes in gray matter that correlate with specific neuropathologies and neurological conditions such as Alzheimer’s disease, schizophrenia and epilepsy. The thickness of the cortex can be a useful measure for understanding disease progression, for identifying affected brain regions and possibly for assessing treatment. It can also be an interesting metric for studying how the normal brain develops and ages.

Using current magnetic resonance imaging techniques, it is possible to measure cortical thickness in-vivo. Anatomical images can be acquired routinely with 1 mm^3^ resolution and optimal contrast between grey and white matter using specially tailored sequences e.g. (Deichmann et al., 2000; Deichmann et al., 2004).With such images the cortical sheet can be clearly identified and the thickness measured manually on image slices. However, since the cortex has a complex three-dimensional structure this task is tricky and labor-intensive. The emergence of image processing methods to automatically calculate cortical thickness over the whole brain in MRI is therefore not surprising (Fischl and Dale, 2000; Jones et al., 2000; MacDonald et al., 2000; Miller et al., 2000; Zeng et al., 1999).With such techniques, it has become more feasible to study large populations of subjects and make comparisons between patients and controls. For example, studies using automated thickness measurements have demonstrated regional patterns of age-associated cortical thinning (Salat et al., 2004) and longitudinal changes in cortical thickness in children (Sowell et al., 2004). Automated methods have also been used to improve detection of focal cortical dysplasia lesions (Antel et al., 2002), to study Huntingdon’s Disease (Rosas et al., 2002), schizophrenia (Kuperberg et al., 2003; Narr et al., 2005), multiple sclerosis (Sailer et al., 2003), and Alzheimer’s disease (Lerch et al., 2004).

Automated methods for measuring cortical thickness in MRI may be categorized as either surface-based, voxel-based or a mixture of the two. In general, surface-based techniques involve the generation of one or two surface models that are driven by image information and surface geometry to fit the grey and white matter surfaces of the image (Fischl and Dale, 2000; Jones et al., 2000; MacDonald et al., 2000; Miller et al., 2000; Zeng et al., 1999). The thickness of the cortex is then defined at surface points or vertices and is given by some measure of the distance between them. Another type of approach involves extracting only the surface between the grey and white matter, then the thickness values that are determined volumetrically by calculating the normal distance from voxels in the cortex to the surface, are mapped towards the surface (e.g. Lerch and Evans, 2005; Thompson et al., 2005). A variation of this approach involves the construction of image intensity-based metrics as a function of normal distance from the extracted surface from which cortical thickness can be quantified (Miller et al., 2000; Miller et al., 2003). This idea has been extended further by stochastically modeling image intensity as a function of normal distance with cortical thickness being one of the model parameters (Barta et al., 2005).

For methods involving surfaces, the comparison of thickness between subjects usually involves matching the corresponding cortical surfaces. In (Fischl et al., 1999), the modeled surfaces have the topology of a sphere which allows the surfaces from different subjects to be matched in a spherical surface-based coordinate system in which thickness comparisons can be made.

Voxel-based cortical thickness measurements do not require the construction of a three-dimensional surface model. Grey and white matter boundaries are instead defined on the basis of whole voxel information (Hutton et al., 2002; Jones et al., 2000; Yezzi and Prince, 2003). The cortical thickness is then calculated at every volumetric point within the cortex and is based on the length of the trajectory from one boundary to another.

Here we investigate a method for generating voxel-based cortical thickness (VBCT) maps from anatomical MRI. This technique results in maps where each voxel in the grey matter is assigned a thickness value. If required, the VBCT maps from different images and different subjects can be transformed into a standardized space using linear or non-linear spatial normalization procedures such as those implemented in SPM2 (Ashburner and Friston, 1999; Ashburner and Friston, 2005). Regionally specific differences in cortical thickness between subjects or changes in cortical thickness over time can then be compared on a voxel-by-voxel basis. This general approach for comparing VBCT measures across different brain images can be considered to be a complementary technique to voxel-based morphometry (VBM) (Ashburner and Friston, 2000; Good et al., 2001), which compares local grey matter concentration or volume. VBCT maps may be particularly advantageous for the analysis of conditions that are associated with cortical thinning, such as dementia, because the local topography of the grey matter is used to assign an absolute metric to grey matter voxels. In contrast, with VBM local grey matter density or volume will be confounded by how convoluted the brain is in a given region.

In brief, our method takes grey matter (GM), white matter (WM) and cerebrospinal fluid (CSF) probability maps resulting from segmented MR images. Morphological operators are used to extract grey matter whilst preserving the topological details of the sulci. The method for calculating thickness by solving Laplace’s equation for the cortical volume (Jones et al., 2000) has been extended by applying it to sublayers of voxels within the cortex to identify regions of buried cortex ensuring that the thickness of grey matter within sulci is not over-estimated. The Laplace equation is then solved for all voxels in the cortex excluding the ones identified as being within sulci and the directions of trajectories connecting one border to the other are assigned to these voxels. The thickness value at each voxel is then calculated as the sum of the distances from that voxel to the inner and outer borders of the grey matter taking into account sulcal voxels where necessary. Although grey and white matter borders are defined on the basis of whole voxel information, sub-voxel measurements of thickness are possible using sub-sampling and interpolation of the image information.

In this paper, the robustness and reproducibility of the method is investigated by applying it to repeated MRI scans of a single subject from two MRI scanners of different field strengths. The method is also applied to a publicly available simulated data set (Lerch and Evans, 2005), in which the right superior temporal gyri (rSTG) of half of the subjects has been artificially thinned, to show that small focal differences in thickness between two groups of subjects can be detected.

## Materials and methods

The following four sections describe (i) how thickness is defined and calculated, (ii) the procedure used to create the VBCT maps, (iii) the analysis of VBCT maps calculated from repeated MRI scans of a single subject from two MRI scanners and (iv) VBCT analysis of a simulated data set.

### Measuring thickness

**Fig. 1:**
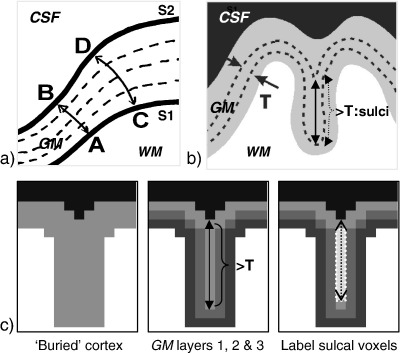
(a) Non-straight line measure of thickness. Laplace's equation is solved for each point between the two surfaces (or boundaries), S1 and S2, resulting in a scalar field describing the smooth transition between them. From this scalar field, the direction of any trajectory from S1 to S2 can be computed. Integrating along any trajectory results in a thickness measure, e.g. from A to B (or B to A) and from C to D (or D to C). (b) Buried cortex where the CSF between the sulci is not resolved. Single layers of GM voxels are successively processed to identify voxels that are within the same layer, but from opposite sulcal banks. These are voxels that have a calculated thickness greater than the expected thickness *T* of a single layer of voxels. (c) Illustration of the steps required to identify buried cortex. Left panel: Schematic representing ‘buried’ cortex and starting point for adding GM layers. Middle panel: GM layers are illustrated in different shades of grey. The thickness calculated for voxels in the third GM layer that also run along the middle of the sulcus will be much greater than the expected thickness *T*. Right panel: Voxels with a calculated thickness greater than *T* are labelled as sulcal voxels.

A variety of distance metrics for measuring cortical thickness have been proposed in the literature (for a comparison, see (Lerch and Evans, 2005)). Mostly these are straight line metrics connecting the inner and outer surfaces of the grey matter for example by the shortest path or the path normal to one of the surfaces. In this work the non-straight line distance proposed by (Jones et al., 2000) is used. This method solves Laplace’s equation to construct trajectories passing through the cortical sheet connecting one surface to the other. A particular advantage of this approach is that for any path or trajectory there is mutual correspondence between the points on the two surfaces regardless of the trajectory’s starting point (Fig. 1a). This cannot be guaranteed when using a straight line distance measure.

The method works by assigning voxels within the GM an arbitrary starting value *B* while voxels on the inner and outer surfaces of the volume are assigned two different boundary conditions or potentials, *B*1 and *B*2 where *B*1 < *B* < *B*2. Laplace’s equation (Eq. 1) is solved for each point within the volume to calculate the scalar field *V*.

(1) $$\nabla^{2}V=\frac{\partial^{2}V}{\partial x^{2}}+\frac{\partial^{2}V}{\partial y^{2}}+\frac{\partial^{2}V}{\partial z^{2}}=0$$

Due to the boundary conditions (*V* equals *B*1 and *B*2 on the inner and outer surfaces respectively), there is a unique solution to this equation which can be found iteratively using a standard relaxation method such as the one described in (Jones et al., 2000). The resulting scalar field makes a smooth transition from one surface to the other. The gradient of the scalar field at each point can then be normalized to produce a tangential vector field **N** (Eq. 2).

(2) $$N=\frac{\nabla V}{||\nabla V||}$$

**Fig. 2:**
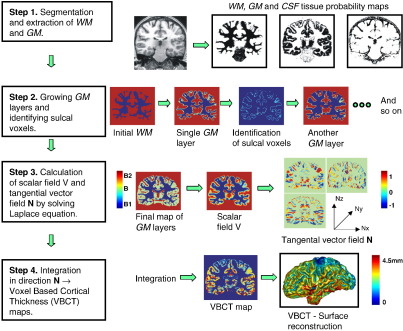
The steps involved in the calculation of VBCT maps.

Integrating in the direction defined by **N** at any point in the volume, from one boundary to the other, provides the length of the trajectory and hence the cortical thickness (as illustrated in Fig. 1a and in steps 3 and 4 of Fig. 2).

### Generating VBCT maps

#### Tissue segmentation

The first step is to segment the MR images into grey matter (GM), white matter (WM) and cerebrospinal fluid (CSF). In this work, two slightly different segmentation methods are performed. The first method is a Bayesian approach combining tissue classification with intensity correction for image inhomogeneities (Ashburner and Friston, 1997) and is implemented in SPM2 (http://www.fil.ion.ucl.ac.uk/spm/software/spm2). This segmentation approach is used in the analysis of the simulated data set. The second method, which is used for within-subject analysis, with repeated MRI scans, is a recent extension of the former segmentation method that also combines a Bayesian tissue classification with intensity correction but performs non-linear tissue probability template matching (Ashburner and Friston, 2005). This method alternates between classification, correction for intensity variations (usually caused by *B*1 inhomogeneity associated with the radio frequency head coil) and non-linear registration to a set of tissue probability templates. With both segmentation methods, voxels in the input MR image are classified according to the intensity value and position with respect to the matched templates. Both methods result in maps of belonging probabilities for GM, WM and CSF in the space of the input data (Fig. 2, step 1). The sum of the tissue probabilities at each voxel adds up to one.

#### Extraction of GM and WM

The GM, WM and CSF probability maps are discretised by assigning to each voxel the tissue class with the greatest probability. To exclude the cerebellum from the analysis, an image of a cutting plane that cuts through the brain stem of the WM probability template image is transformed into the space of the input data and multiplied by the WM probability map separating the WM in the cerebellum from the rest of the brain. The largest connected components resulting from connected component analyses (Thurfjell et al., 1992) of the GM and WM images are selected as the initial maps of GM and WM voxels respectively.

### Preserving cortical topography

If the CSF spaces between narrow sulci are not well resolved, regions of cortex can appear to be ‘buried’ so that the thickness in these regions may be overestimated. This is illustrated in Fig. 1b where the thickness may be incorrectly estimated from the bottom of the sulci at the boundary between WM and GM up to the boundary between GM and CSF. The initial map of grey matter voxels, GM, will include voxels within buried cortex. These need to be identified to preserve the topography of the cortical sheet and ensure that the thickness is not overestimated in these regions. The following section together with Fig. 1c describes this process.

Starting from the initial WM map (Fig. 1c, left panel), layers of isotropic voxels are successively added to surround the WM (Fig. 1c, middle panel). The expected thickness of each single layer of voxels is *T*, where *T* is determined by the voxel size. For example, a single layer of 1 mm^3^ isotropic voxels will result in a layer of thickness ranging from 1 mm up to $\sqrt{3}$ mm depending on the orientation of the layer. Each layer is labeled as GM and its thickness is then calculated by solving Laplace’s equation within the layer, computing the scalar field *V* (Eq. 1) and the tangential vector field **N** (Eq. 2). For each single layer, the calculated thickness is compared with the expected thickness *T*. In Fig. 1c, middle panel, the first two GM layers will not contain any voxels with a thickness greater than *T*. However, as can also be seen in Fig. 1c, middle panel, the thickness calculated for voxels in the third GM layer that also run along the middle of the sulcus will be much greater than *T*. These voxels are labeled as sulcal voxels (Fig. 1c, right panel, voxels highlighted in middle of sulcus). This is because these voxels are in contact with GM voxels within the same layer but from an opposite sulcal bank (Fig. 1b and Fig. 1c right panel). As can be seen in Fig. 1c, the location of the CSF space within the sulcus is ambiguous. It is therefore considered as being in the centre of the sulcal voxels (black dotted line in Fig. 1c, right panel). Sulcal voxels are used to indicate where the boundary is between GM and CSF and are therefore excluded from the GM map at this stage. However, in the final integration step which calculates the VBCT maps, the path through the sulcal voxels is included in the thickness calculation as described in *Calculation of VBCT maps*.

Layers are added to the WM until the outer limit of the GM is reached or a maximum number of layers have been added. The maximum number of layers prevents too many layers growing into the brain stem and sub-cortical structures. In this work, the maximum number of layers has been empirically defined to be the number of voxel layers not exceeding approximately 10 mm and is a maximum value which is reasonable to ensure that all cortical GM was included (since cortical thickness should not exceed around 5 mm). These steps result in a map identifying which voxels are in narrow sulci and a final map of GM formed by combining the processed GM layers for which the thickness can be calculated (Fig. 2, step 2).

#### Calculation of VBCT maps

The VBCT maps are calculated for the final maps of GM (i.e. excluding the sulcal voxels). This is achieved by solving Laplace’s equation at every point in the final GM map to calculate the normal direction **N** for every voxel in the GM (Fig. 2, step 3). The next step is to calculate the total length of the path between two boundaries of the GM, by integrating along the trajectories described by **N** for each voxel in the GM. This is achieved by considering each voxel in turn as a start point P1 along the trajectory, with coordinates (*x, y, z*). From the start point, a step of length *stepsize* is taken in the direction defined by the normal at P1, i.e. **N**(*x, y, z*). The new position along the trajectory will then be point P2 with coordinates (*x* + Δ*x,y* + Δ*y, z* + Δ*z*) where Δ*x* = **N**_*x*_(*x, y, z*) × *stepsize*, Δ*y* = **N**_*y*_(*x, y, z*) × stepsize and Δ*z* = **N**_*z*_(*x, y, z*) × *stepsize*. At point P2, the normal direction has a new value given by **N**(*x* + Δ*x,y* + Δ*y,z* + Δ*z*). This process continues until a boundary or sulcal voxel is reached. It is then repeated for *stepsize* multiplied by −1, starting from point P1, to integrate along the trajectory in the opposite direction. The resulting path length is the total sum of the steps from each voxel to both boundaries. For sulcal voxels, **N** has not been defined therefore the normal direction through the preceding voxel is used and the additional path length through this voxel is added on to the total path length. The resulting VBCT maps can be displayed volumetrically or on a surface reconstruction (Fig. 2, step 4).

#### Smoothing and thresholding VBCT maps

There are several reasons for smoothing VBCT maps. Firstly, an estimate of cortical thickness is calculated for every voxel in the grey matter resulting in relatively noisy values because Laplace’s equation is solved on a discrete grid with a finitely sized integration step. Smoothing the VBCT maps will therefore improve the signal to noise ratio and make any subsequent voxel-by-voxel analysis comparable to a region of interest approach because each voxel in the smoothed VBCT maps will contain the average thickness around the voxel. If comparing VBCT maps from different subjects, smoothing compensates for residual anatomical variability after spatial normalization and renders the data more normally distributed increasing the validity of any statistical parametric test. The size of the smoothing kernel should be chosen to reflect these points and when possible it should be comparable to the size of the expected regional differences. In more general terms, the reasons for smoothing are similar to those for smoothing grey matter probability maps when doing VBM (Ashburner and Friston, 2000). However, when investigating within subject cortical thickness, it is also important to keep smoothing to a minimum so that any abrupt changes in thickness that may occur at the boundaries between cortical areas are not obscured.

A cortical thickness value is estimated for all voxels classified as GM so sub-cortical structures such as basal ganglia and extra-cortical tissue such as meninges may be included in the VBCT maps. However, only voxels in the grey matter sheet are of interest, therefore means and standard deviations are calculated after thresholding the smoothed VBCT maps at 5 mm. This threshold value was chosen as the upper limit for cortical thickness values reported in the literature.

#### Implementation and display

The routines described above are fully 3-dimensional, automated and implemented in Matlab 6.5 (The Mathworks Inc., Natick, MA) and C. After segmentation, tissue probability maps can be sub-sampled for the subsequent steps involved in the calculation of VBCT maps. This increases processing time but allows smaller structures, particularly narrow CSF spaces, to be resolved. The integration used to calculate thickness can also be performed with a sub-voxel step-size. The final VBCT maps are written out in the same space and at the same resolution as the original input data.

The VBCT maps can be viewed as three-dimensional volumes or used to colour a surface rendering of the brain. In this paper, the surface renderings have been generated in Matlab by extracting the isosurface representing the mid-point of the *GM*. The same *GM* mid-point has also been inflated using BrainVoyager 2000 (Brain Innovation, Maastricht, The Netherlands). The smoothed VBCT maps are then sampled at each of the resulting surface vertices and used to colour the surface renderings.

### Within Subject Comparison of VBCT maps

The purpose of this study was to investigate the robustness and reproducibility of VBCT analyses for repeated MRI scans of the same subject from two different MRI scanners.

#### Data acquisition

Anatomical images of one healthy volunteer were acquired on two different Siemens MRI scanners (Siemens Medical Systems, Erlangen, Germany). Six image volumes were acquired on a 3 T Allegra head only scanner with head coil for transmission and reception. Two days later, six images were acquired on a 1.5-T Sonata whole body scanner with a whole body coil for transmission and an eight channel phased-array head coil for signal reception. On both scanners, not all of the images were acquired within the same session so much care was taken to position the subject in the same way for each scan.

On both scanners, the 3D MDEFT sequence was used (Ugurbil et al., 1993) with asymmetric positioning of the inversion pulse within the preparation part of the sequence (Deichmann et al., 2004). For the following sets of acquisition parameters, TI is the total duration of the preparation part, τ1 is the delay between the saturation and the inversion. At 1.5 T, the acquisition parameters were: TR/TE/TI = 12.24/3.56/530 ms, bandwidth = 106 Hz/Pixel, flip angle = 23°, τ1/TI = 42%. At 3 T, the acquisition parameters were: TR/TE/TI = 7.92/2.4/910 ms, bandwidth = 195 Hz/Pixel, flip angle = 15°, τ1/TI = 50%. The acquisition parameters common to both scanners were matrix = 256 × 224, FOV = 256 × 224 mm, number of slices = 176, acquisition orientation = sagittal, slice thickness = 1.0 mm, voxel size = 1.0 mm^3^, total duration = 12 min.

#### Calculation and comparison of VBCT maps

The MRI volumes were segmented using the combined method for Bayesian tissue classification and non-linear tissue template matching (Ashburner and Friston, 2005). The segmented data (with 1 mm voxel resolution) were sub-sampled at 0.5 mm and VBCT maps were calculated for each MRI volume as described above. An integration step-size of half the sub-sampled voxel size, i.e. 0.25 mm, was used to calculate the thickness.

The segmentation method also yields the non-linear spatial transformation parameters matching the input image to the tissue probability templates. These were applied to the calculated VBCT maps to transform them into MNI space (Evans et al., 1993). In this step, nearest neighbour interpolation was used to preserve the calculated thickness values.

The spatially normalised VBCT maps were smoothed using a three-dimensional Gaussian smoothing kernel with FWHM = 3 mm. A smoothing kernel of this size was chosen to reflect the average thickness of the cortex and to account for any small discrepancies in spatial normalisation. Gaussian smoothing slightly reduces the values in the VBCT maps because it performs a weighted averaging over all voxels included by the Gaussian kernel and the values of some of those voxels are zero if they do not consist of grey matter. This effect was corrected for by dividing the smoothed VBCT maps by a binary mask of each VBCT map which has had the same smoothing applied.

Maps of the mean and standard deviation of the VBCT maps (smoothed, normalised and corrected for smoothing effects), across the six scans for each scanner were calculated and displayed on renderings of the cortical surface and the inflated cortical surface. A standard deviation map was also calculated across all twelve VBCT maps. Histograms of cortical thickness values were generated for each VBCT map. A statistical comparison of the VBCT maps calculated for each scanner was performed using a two-sample *t*-test in SPM5 (http://www.fil.ion.ucl.ac.uk/spm/software/spm5/). *T*-scores were calculated and p-values generated on the basis of a family-wise error correction for multiple comparisons. Voxels with *p* < 0.05 were defined to have a significantly different thickness calculated for one scanner compared with the other.

### VBCT analysis of a simulated population

The purpose of this study was to determine whether the VBCT analysis was able to detect a small regional change in thickness in a simulated population study.

#### Simulated data

The publicly available simulated population data comprises 50 MRI image volumes which have already been transformed into stereotactic space and segmented into different tissue types (Lerch and Evans, 2005). In this dataset, the right superior temporal gyri (rSTG) of 25 of the images have been artificially thinned to create a patient group. The authors describe that one layer of voxels in the rSTG was removed which should result in approximately 1 mm difference between the cortical thickness of the two groups in this region.

#### Calculation of VBCT maps

The simulated data were already segmented but required further segmentation to separate the brain from extra-cortical tissue and the cerebellum. Segmentation based on (Ashburner and Friston, 1997) was used rather than the more recent method (Ashburner and Friston, 2005) for two reasons. Firstly, the simulated data were already transformed into stereotactic space, so that the spatial normalization step of the more recent segmentation algorithm was redundant. Secondly, the voxel values of the segmented simulated data were not normally distributed, rendering this data invalid for the more recent segmentation approach.

After the additional segmentation, VBCT maps were calculated as described above for the 50 data-sets. The segmented data were analysed at the resolution of the input data, (i.e. 1 mm) and the integration step-size for calculating the thickness was 0.5 mm. Three-dimensional Gaussian smoothing with FWHM = 12 mm was applied to each VBCT map. A kernel size of 12 mm was chosen as a trade-off between looking for changes in cortical thickness, of the order of a few millimetres, while allowing for anatomical variability which can be between 1 and 2 cm.

#### Statistical comparison of VBCT maps

To identify voxels for inclusion in the statistical analysis and to determine the number of multiple comparisons, a smooth GM mask of the brain was created by performing a logical ‘OR’ over the VBCT maps followed by morphological opening (Gonzalez and Woods, 1992), smoothing and thresholding. A group comparison of the smoothed VBCT maps was performed using a simple linear regression model in SPM2. *T*-scores were calculated for the model fit and p-values generated on the basis of a family-wise error correction for multiple comparisons over the GM. Regions of the cortex comprising voxels with *p* < 0.05 were defined to be significantly thinner in one group compared with the other. The significantly thinner region was used to define a region of interest within which the mean and standard deviation of the difference between the means of the two groups of data were calculated.

## Results

### Within-subject comparison of VBCT maps

**Fig. 3:**
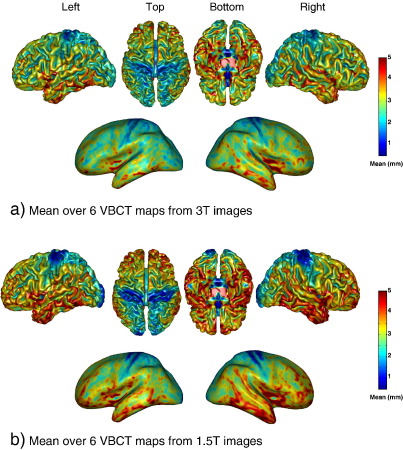
Mean cortical thickness over the six VBCT maps calculated for images acquired on the 3 T scanner (a), and on the 1.5 T scanner (b). In the second and fourth rows, the mean thickness is shown on the cortical surface after inflation. The surface colouring corresponds to the mean thickness in mm. (For interpretation of the references to colour in this figure legend, the reader is referred to the web version of this article.)

Fig. 3 shows surface renderings of the mean of the six VBCT maps from the 3 T scanner (a), and the 1.5 T scanner (b). The colouring of the surfaces corresponds to the mean thickness in millimetres as indicated by the colour scale. The mean VBCT maps from the two different scanners both show an overall increase in thickness from the posterior to the anterior regions of the brain. The thinnest regions are around the occipital area and within the central sulcus. The thickest regions are the superior frontal area and around the temporal lobes. The inflated surfaces indicate that thickness is greater at gyral ridges than within the sulci. This spatial organization of thickness is consistent with the literature (Zilles, 1990) with the exception of the anterior bank of the central sulcus which is reported to be much thicker than the posterior bank (Brodmann , 1908; Von Economo and Koskinas, 1925). In these results, both the anterior and posterior banks of the central sulcus are amongst the thinnest regions of the brain, and this is more apparent in the 1.5 T VBCT maps. This is addressed in the Discussion.

**Fig. 4:**
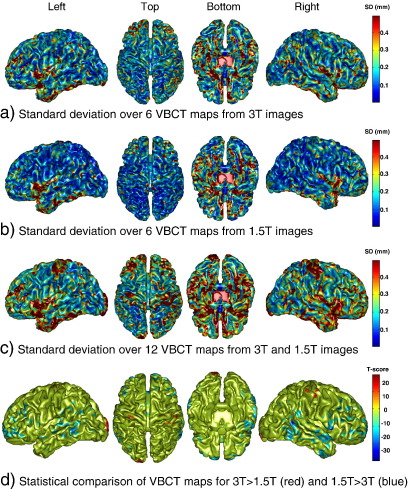
Standard deviation of the cortical thickness over the six VBCT maps calculated for images acquired on the 3 T scanner (a), on the 1.5 T scanner (b) and over all twelve scans (c). The surface colouring corresponds to the standard deviation of the thickness in mm. (d) Statistical comparison between VBCT maps calculated for 3 T greater than 1.5 T scanner (positive T-scores in red) and 1.5 T greater than 3 T (negative T-scores in blue). T-scores are thresholded at ± 11.295 which corresponds to a corrected p-value of 0.05. (For interpretation of the references to colour in this figure legend, the reader is referred to the web version of this article.)

Fig. 4 shows surface renderings of the standard deviation of the six VBCT maps from the 3 T scanner (a), the 1.5 T scanner (b) and over all twelve VBCT maps (c). The colouring of the surfaces corresponds to the standard deviation of thickness in millimetres as indicated by the colour scale. The within-scanner standard deviation of the VBCT maps (Figs. 4, a and b) show that over most of the brain, the standard deviation is less than 0.25 mm. Regions of higher standard deviation (up to 0.4 mm) appear to be randomly scattered or correspond to thicker regions such as the superior frontal area and the insular cortex. The standard deviation of the VBCT maps from both scanners (Fig. 4c) indicates higher values in the region of the central sulcus, the superior frontal area and around the temporal lobes corresponding to the variation in thickness between the means for the two scanners as seen in Fig. 3. Fig. 4d shows the results of the statistical comparison between the VBCT maps calculated for each scanner. The maps of *T*-scores were thresholded at a value of ± 11.295 which corresponds to a corrected p-value of 0.05. Positive *T*-scores (in red) correspond to voxels where the thickness is significantly greater for images from the 3 T compared with the 1.5 T scanner. These voxels occur in the region of the left and right central sulcus and the left occipital pole. Voxels with a significantly greater thickness for images from the 1.5 T compared with the 3 T scanner (negative *T*-scores in blue) are located in superior frontal and temporal regions. These results are in concordance with the differences observed in the mean and standard deviation maps.

**Fig. 5:**
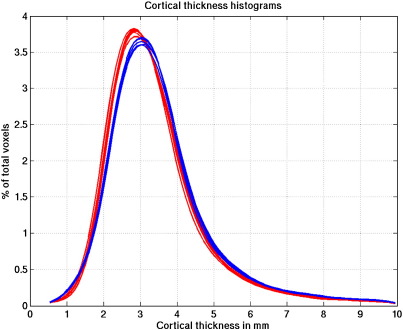
Histograms of cortical thickness values where numbers of voxels are shown as a percentage of the total number of voxels having thickness values up to 10 mm. Data from the 3 T scanner are shown in red and from the 1.5 T scanner in blue. (For interpretation of the references to colour in this figure legend, the reader is referred to the web version of this article.)

**Fig. 6:**
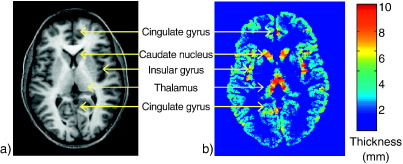
Slice through MRI (a) and corresponding VBCT map (b). The VBCT map slice shows regions where the thickness values are greater than 5 mm. These regions correspond to sub-cortical structures and some voxels in the insular and cingulate cortex.

Fig. 5 shows histograms of cortical thickness values for the 3 T scanner (red) and the 1.5 T scanner (blue) where numbers of voxels are shown as a percentage of the total number of voxels having thickness values up to 10 mm. Around 85% of the voxels have thickness values less than 5 mm. There is clear consistency between the two sets of six within-scanner VBCT map histograms but discrepancy between the results for the 1.5 T and 3 T scanners. The mean thickness over the brain (calculated to include only voxels with thickness less than 5 mm) is 3.1 mm for both 1.5 T and 3 T images. The mean of the standard deviation map calculated for the within-scanner VBCT maps is 0.2 mm for both scanners. The mean of the standard deviation map calculated for VBCT maps from both scanners is 0.3 mm. Fig. 6 shows a slice through a VBCT map showing an example of regions where the cortical thickness is greater than 5 mm. In this slice, these regions mostly correspond to sub-cortical structures and voxels in the insular and cingulate cortex.

### VBCT analysis of a simulated population

**Fig. 7:**
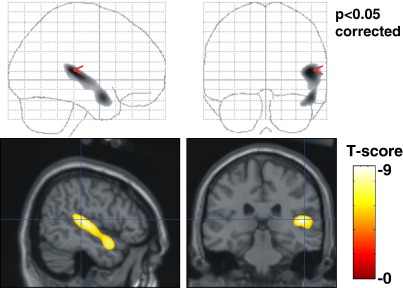
Map of *T*-scores indicating voxels that are significantly thinner in the group where the rSTG had been artificially thinned (*p* < 0.05 corrected). The top row shows a maximum intensity projection of the *T*-scores. In the bottom row the *T*-scores are overlaid on the MNI/ICBM canonical brain image.

Fig. 7 shows the map of *T*-scores indicating which voxels are significantly thinner in the group of data for which the rSTG had been artificially thinned (thresholded at *p* < 0.05 corrected for multiple comparisons). In the top row, the maximum intensity projection of the *T*-scores is shown on the glass brain and in the bottom row, sagittal and coronal sections through the *T*-scores are overlaid on the MNI/ICBM canonical brain image (Evans et al., 1993). 8726 voxels within the rSTG survived a family-wise error correction over the grey matter of *p* < 0.05. No voxels were significantly thicker in the artificially thinned rSTG group. The mean(standard deviation) of the difference in thickness between the two groups of VBCT maps within the significantly thinner rSTG region = 0.68(0.43) mm.

## Discussion

In this work we have investigated a method for generating VBCT maps, to determine its robustness, reproducibility, and ability to detect differences in cortical thickness between two groups of subjects. To do this, VBCT analyses were performed in a series of repeated MRI scans from a single subject acquired on two MRI scanners and in a simulated population study.

### VBCT versus surface-based methods

Currently, the majority of published studies using automated methods for measuring cortical thickness have been carried out using surface-based techniques for which the reliability has been demonstrated (Han et al., 2006; Lerch and Evans, 2005). With these methods, the thickness is calculated at each point on the extracted cortical surface and surface-based smoothing is applied to the results. The benefit of this type of smoothing should be that it prevents the problem of averaging across different banks of sulci and gyri. However, this can only be ensured if the surface has been accurately extracted. Depending on the constraints associated with the method, it may be difficult to correctly fit a deformable surface model into deep sulci or buried cortex. Furthermore, such constraints may confound the analyses of abnormally structured brains. In this work, isotropic Gaussian smoothing has been applied to the cortical volume. Although this is a simple, practical way to improve the signal-to-noise ratio of the cortical thickness measurements and to account for anatomical variability, it is also disadvantageous due to the highly curved nature of the cortex. Performing anisotropic smoothing on the volume, for example in the direction orthogonal to the normal trajectories through the cortex, could provide a more natural representation of the cortical mantle and should be an area of further work.

An advantage of the VBCT method presented here is that topological constraints are imposed using a layer-growing process. In areas of the brain where there are thin regions of CSF between the sulcal banks, skeletonizing (Gonzalez and Woods, 1992) the CSF may be an interesting alternative method for identifying deep sulci. However, the method presented here attempts to account for buried cortex and touching sulcal banks for sulci of sub-voxel width, even when there is no information about where the CSF lies. The voxel-based process means that a thickness value is calculated at every voxel in the cortex resulting in more than one measure of thickness for each trajectory or path connecting one surface to the other. These multiple measurements could be used to provide an indication of the signal to noise ratio which may be advantageous when comparing thickness across different scans. However, there are some clear advantages to surface-based comparisons of cortical thickness compared to volumetric comparisons. First, assuming that the surface has been accurately extracted, one can be sure that one point in the cortex is being compared with another. In contrast, when comparing volumes this feature is dependent on how well the data has been spatially normalized. Secondly, using volumetric comparisons, thicker areas of cortex will contain more voxels with a given thickness value so that comparisons in these regions will be inherently biased. Finally, since there are fewer vertices in a cortical surface compared with voxels in a cortical volume, the number of multiple comparisons is reduced. These last two points may be tackled by performing smoothing or averaging within the volume so that VBCT analyses are based on a smaller number of multiple comparisons.

Since all voxels classified as grey matter are included in the analysis, sub-cortical and extra-cortical structures may be included. Although calculating the thickness of such structures is not necessarily meaningful since they do not have the sheet-like structure of the cortex, this does not affect the results of voxel-wise comparisons of thickness in the cortex. If required, a volumetric atlas could be used to exclude the unwanted structures and a deformable surface model could be incorporated into the VBCT framework to impose further topological constraints.

### Within-subject comparison of VBCT maps

Cortical thickness values reported in the literature mostly range from a mean thickness over the whole brain of around 2.5 mm up to 3 mm. For the subject studied in this experiment, we estimated a mean thickness over the brain of 3.1 mm. When comparing mean thickness over the brain calculated using different methods (in vivo and post mortem) it is important to consider several things. Firstly, the amount of cortical and non-cortical GM included in the calculation should be known. In this work, the approach has been to calculate the mean of voxels with a thickness value less than 5 mm. Secondly, current opinion seems to suggest that thickness measurements made on post-mortem brains are not directly comparable with in vivo techniques using MRI because stained brain sections exhibit different structural properties from in vivo brain MR images. Furthermore, it has been demonstrated that thickness values are dependent on the metric used to estimate them (Lerch and Evans, 2005). The VBCT maps reported in this study lean slightly towards the higher end of reported values. This is attributed to using the method based on solving Laplace’s equation, which gives larger values since it calculates a non-straight line distance (Jones et al., 2000; Lerch and Evans, 2005). In general, other factors that will affect the magnitude of the estimated cortical thickness in MRI are the quality of the original MRI, the reliability of the segmentation method and the amount of smoothing applied to the thickness measurements.

In the results presented here the anterior bank of the central sulcus is one of the thinnest regions of the brain which is inconsistent with histological studies indicating that it is one of the thickest (Brodmann , 1908; Von Economo and Koskinas, 1925). There is also a significant difference between the results for the two scanners in the central sulcal region, with the thickness calculated for 3 T being greater than that for 1.5 T. There are several factors which may contribute to this disagreement. Firstly, the thickness calculation requires an accurate map of the boundaries of grey and white matter. These boundaries are determined by discretising probability maps resulting from a tissue segmentation step. On closer inspection of the segmentation results, particularly from the 1.5 T data, it could be observed that some voxels in the anterior bank of the central sulcus were assigned a higher probability of being white matter than grey matter. This ambiguity may be due to the difficulty of imaging this particular part of the brain towards the top of the head. This region is most likely to suffer from inhomogeneities of the radio-frequency (RF) coil in both transmit mode, giving rise to different image intensity scaling, and receive mode which can affect tissue contrast. This problem, which can be particularly apparent in images acquired using a phased-array head coil, has been reported in (Deichmann et al., 2004). Further more, the bright fat signal from the scalp can lead to artefacts in the presence of subject motion (Howarth et al., 2006). Therefore alternative MR image acquisition sequences or segmentation methods may be required to accurately delineate the cortex in this part of the brain. Over the rest of the brain, the spatial pattern of thickness is consistent with the literature and the mean of the between-scan, within-scanner standard deviation is 0.2 mm. The standard deviation over the VBCT maps from the two scanners together is higher (up to 0.4 mm) in the central sulcus, insular cortex and inferior temporal lobes. This indicates that although the same acquisition sequence was used on each MRI scanner, there are small but significant differences in the segmented versions of the 1.5 T and 3 T images.

### VBCT analysis of a simulated population

A further purpose of this study was to determine whether a voxel-based approach for measuring cortical thickness could detect a small (∼ 1 mm) regional change in thickness in a simulated population study. The VBCT analysis was able to detect a large contiguous area within the rSTG (*p* < 0.05 corrected) with a mean difference in thickness of 0.68 mm. These results were attained using a Gaussian smoothing kernel of 12 mm, which was chosen to account for anatomical variability. However, since the data are simulated, it would in general be possible to determine the best smoothing kernel for this data set given a precise map of the artificially thinned region. The data were analysed without any sub-sampling suggesting that it may be possible to detect a smaller difference (< 1 mm) with sub-sampled data.

The analysis of this population data set differs from that of the repeated MRI scans, because the input data had previously been spatially registered and segmented using different methods as described by the dataset authors. In the presented method for measuring VBCT, an important factor is that the thickness is calculated on images in their native space and spatial transformations to the VBCT maps are made subsequently. We therefore do not make any comparisons between the single subject results and the simulated population results, but we do intend to pursue a similar group analysis on our own MRI data.

### VBCT analysis versus VBM

For comparative brain morphometry, we propose that VBCT evaluations be considered as an analysis methodology that is complementary to VBM analysis of structural image data. With VBM, spatially normalized probability maps of grey and white matter are statistically compared. During spatial normalization, the volumes of certain brain regions may be stretched or shrunk. To account for this, a modulation step is performed that involves multiplying the normalized grey matter by the relative regional volume before and after normalization so that the total amount of grey matter signal in the normalized region is preserved. This is commonly referred to as optimized VBM (Good et al., 2001). The analysis of data after performing this modulation step tests for regional differences in grey matter volume, whereas without the modulation step differences in grey matter concentration are being tested. In contrast, VBCT maps are calculated using segmented MR images in the native space of the subject’s original MRI assigning an absolute measure of cortical thickness to each grey matter voxel. When the VBCT maps are spatially normalized these absolute thickness values do not change. It is therefore possible to detect absolute differences in thickness using VBCT analyses. The relationship between thickness and local grey matter density or volume has not been established and will probably depend on the pathology or state associated with the morphometric changes being studied. For example, changes in local cortical atrophy may be more clearly defined using VBCT maps than VBM in regions of the brain where the cortex is more convoluted. A comparison between VBM and the analysis of VBCT maps will be the focus of further studies.

## Conclusion

This work demonstrates the feasibility of using a voxel-based method to automatically calculate the cortical thickness over the whole brain. The calculated thickness values fall within the range of values reported in the literature using different techniques. The spatial relationship between the thicknesses of different regions is also consistent with the literature except in the region of the central sulcus. For single subject images acquired on the same scanner, the VBCT method has proved to be robust. The results of the simulated population study show that a difference in thickness of the order of 1 mm can be detected easily suggesting that the analysis of VBCT maps is a useful morphometric tool.
